# A bio-fabricated tesla valves and ultrasound waves-powered blood plasma viscometer

**DOI:** 10.3389/fbioe.2024.1394373

**Published:** 2024-04-24

**Authors:** Wenqin Chen, Mao Xia, Wentao Zhu, Zhiye Xu, Bo Cai, Han Shen

**Affiliations:** ^1^ Department of Clinical Laboratory, Nanjing Drum Tower Hospital, Affiliated Hospital of Medical School, Nanjing University, Nanjing, China; ^2^ School of Environment and Health, Jianghan University, Wuhan, China

**Keywords:** biofabrication, tesla valves, ultrasound wave, viscosity measurement, coagulopathy

## Abstract

**Introduction:** There is clinical evidence that the fresh blood viscosity is an important indicator in the development of vascular disorder and coagulation. However, existing clinical viscosity measurement techniques lack the ability to measure blood viscosity and replicate the *in-vivo* hemodynamics simultaneously.

**Methods:** Here, we fabricate a novel digital device, called Tesla valves and ultrasound waves-powered blood plasma viscometer (TUBPV) which shows capacities in both viscosity measurement and coagulation monitoring.

**Results:** Based on the Hagen-Poiseuille equation, viscosity analysis can be faithfully performed by a video microscopy. Tesla-like channel ensured unidirectional liquid motion with stable pressure driven that was triggered by the interaction of Tesla valve structure and ultrasound waves. In few seconds the TUBPV can generate an accurate viscosity profile on clinic fresh blood samples from the flow time evaluation. Besides, Tesla-inspired microchannels can be used in the real-time coagulation monitoring.

**Discussion:** These results indicate that the TUBVP can serve as a point-of-care device in the ICU to evaluate the blood’s viscosity and the anticoagulation treatment.

## Introduction

Hemodynamics constitutes the comprehensive investigation of fluid dynamics within the circulatory system (Jain et al., 2016; Zhang et al., 2022; Tyagi et al., 2022). The precise characterization of flow behaviors plays a pivotal role in the realms of diagnosing and treating conditions associated with the circulatory system (Ferro et al., 2004; Mandal, 2005; Foley et al., 2012; Chen et al., 2023). Viscosity is possibly the most important parameter to characterize the different phenomena observed in blood flow (Bodnár et al., 2011; Rubio et al., 2022; Jędrzejczak et al., 2023). Usually, the increase of blood viscosity is regarded as the risk for the abnormally elevated blood flow resistance, such as in chronic vascular disease, and endothelial dysfunction (Valeanu et al., 2021; Al-kuraishy et al., 2022; De Leonardis et al., 2022; Wen et al., 2022). Acute physical exercise even causes a 10% increase of blood viscosity (El-Sayed et al., 2005; Connes et al., 2012). Besides, acute assessment of coagulopathy is important for the clinical judgement of anticoagulation drug treatment in diseases like thrombosis or bleeding (Ganter and Hofer, 2008; Carroll et al., 2009; Munoz et al., 2009; Chen et al., 2022). However, current viscometers are limited in their singular focus on viscosity measurements and lack the ability to replicate the *in vivo* hemodynamics accurately (Saha et al., 2021; Fuiano et al., 2022; Yi et al., 2022). Thus, there is a critical need to develop new strategy that enables rapid measurements of blood viscosity and continuous monitoring the coagulopathy simultaneously.

Inspired by Tesla valves, invented by Nikola Tesla, here we design a blood plasma viscometer that can also monitor the coagulopathy. Tesla-inspired microchannels not only can be used as fluidic diodes that allow fluid to pass easily in forward direction while resist the fluid to flow in the reverse direction (Nguyen et al., 2021; Bohm et al., 2022; Li et al., 2023), also can be employed in designs for real-time flow monitoring (Groisman and Quake, 2004; Hong et al., 2004; Hossain et al., 2010; Zhang et al., 2016; Ting et al., 2019; Wang et al., 2022). With the constant pressure driven, the flow shows stable flowing velocity, underscoring its potential for deployment in capillary-type viscometer (Kang et al., 2019; Bhattad, 2023). In addition, the Tesla-like microchannel can mimic the physiological shear rates in blood flows, providing a more faithful platform for analyzing the coagulopathy. Notably, the conventional flow driver in microfluidic viscometers, typically a peristaltic pump, is both cost-intensive, and space-, blood-consuming, showing difficulty in the point-of-care testing (Leach et al., 2003; Xie et al., 2004; Collins et al., 2016; Davis et al., 2021). Thus, a miniaturized and user-friendly platform is supposed to be developed for the microfluidic pumping in the clinic.

Acoustic streaming implemented in microfluidics has triggered various applications, such as mixing, particle manipulation, droplet manipulation, and flow control (Ding et al., 2013; Guo et al., 2015a; Guo et al., 2015b; Guo et al., 2015c; Collins et al., 2017; Hu et al., 2020; Ao et al., 2022; Zhang et al., 2022; Durrer et al., 2022; Wu et al., 2022; Wu et al., 2023a; Wu et al., 2023b; Del Campo Fonseca et al., 2023; Xu et al., 2023). Among these applications, acoustic streaming-induced fluid pumping shows the excellent properties including miniaturization, contactless, precise, stability, and tunability, which has been integrated into the point-of-care usages (Du et al., 2009; Dentry et al., 2014; Huang et al., 2014; Wu et al., 2019; Cai et al., 2020). These features indicate the great potential of acoustic streaming-induced pumping integrated into the designated microchannel. Thus, the integration of acoustic streaming and the Tesla-like microchannel would result in a new blood viscometer that has real-time monitoring functions.

**FIGURE 1:**
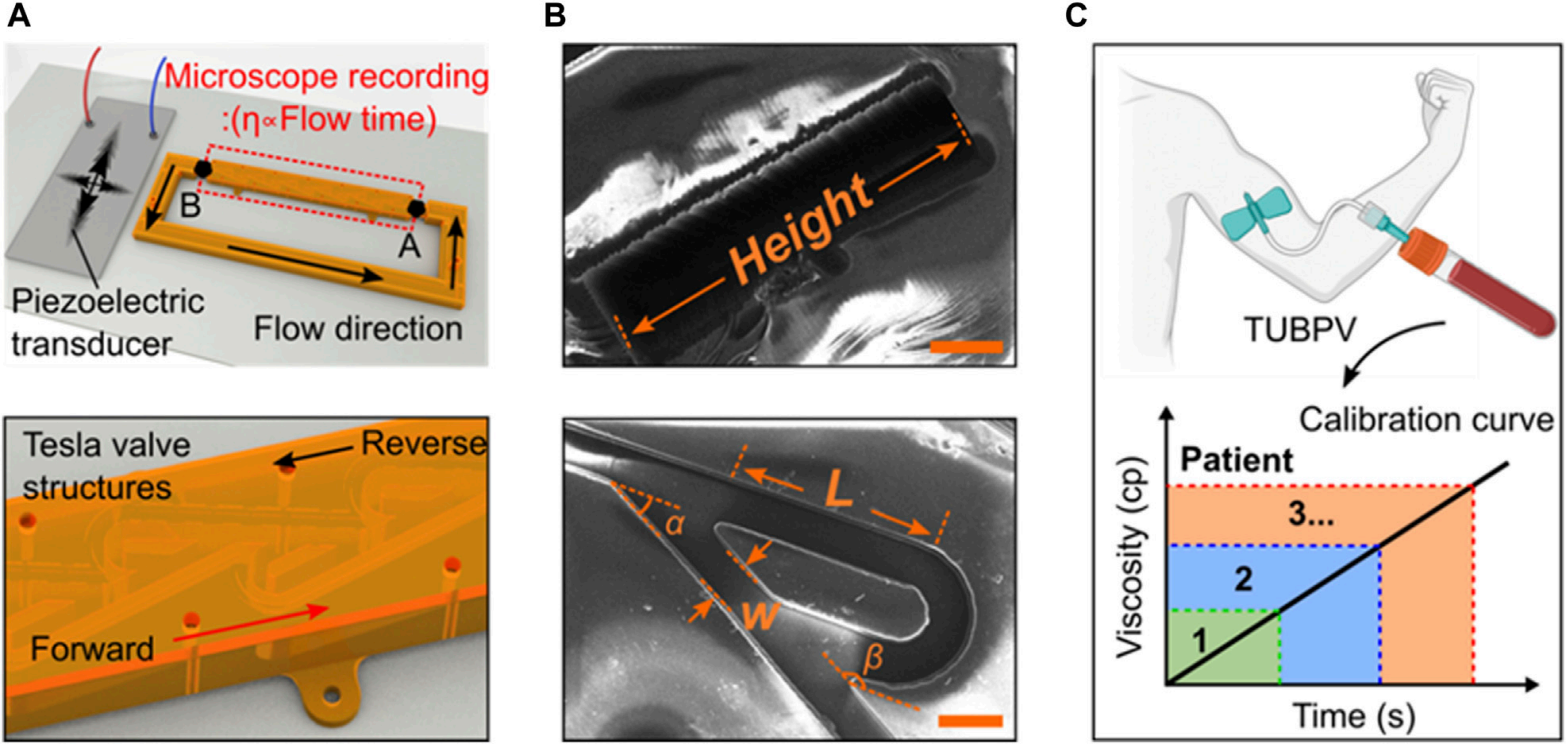
Design and working principle of the blood viscometer. **(A)** Schematics of blood viscometer, including two key components, Tesla valves and ultrasound wave generator. The viscosity of liquid can be calculated by the flowing time from position A to **(B) (B)** The scanning electron microscope (SEM) images showing the design of Tesla valve. W-valve width, L-length of the valve straight segment, α-leaving angle, β-returning angle (Scale bar: 200 μm). **(C)** Schematics of the Tesla valves and ultrasound waves-powered blood plasma viscometer (TUBPV) for measuring the viscosity of patient blood plasma.

In this work, we establish a simple and reliable platform for measuring blood plasma viscosity and monitoring coagulopathy, as shown in Figure 1A. This platform consists of the Tesla valve-based microchannel and ultrasound wave generator. The mechanism of viscosity measurements is similar to that in the capillary-type viscometer. The flow is driven by the acoustic streaming from the ultrasound-structure interaction. Besides, the flow direction is regulated by the Tesla-like structure’s fluid diode. Further, we demonstrate that the viscometer capability based on the Tesla valves and ultrasound waves. Benefited from the controllability of the ultrasound waves, the flow behavior can be programmed and further used to mimic the coagulopathy. These results indicate that the significant potential in the point-of-care blood viscosity and coagulopathy management.

## Results

**Device design and working principle.** The Tesla valves and ultrasound waves-powered blood plasma viscometer (TUBPV) consists of a piezoelectric transducer and a channel with Tesla valve structures (Supplementary Figure S1), showing the stable, fast, disposable, small-volume, and precise measurement properties. This capillary-type viscometer supports visual measurements of liquids’ viscosity proportional to the flowing time under a constant distance, referring to the following Hagen-Poiseuille equation (Prusa, 2008):

$$\eta =\frac{\pi r^{4}∙∆P}{8LV}\left(T_{B}-T_{A}\right)$$ (1)

where η is the liquid viscosity, r is the channel radius, ∆P represents the pressure inside the channel, L is the length from position A to B, V means the liquid volume, and $\left(T_{B}-T_{A}\right)$ is the flowing time. Notably, the pressure is induced and well-controlled by the ultrasonic radiation, driving the liquid movement (Supplementary Movie S1). Combining with the regulation of Tesla valve conduit, the liquid driven by stable ultrasound waves showed the unidirectional and rapid flow motion. The Tesla valve conduit was fabricated by 3D printer and disposable with zero clean up, enabling to load liquid sample as low as 200 μL (Figure 1B). Benefitting from the ultrahigh speed imaging of the charge-coupled device (CCD), the flowing time can be precisely recorded, resulting in the high accuracy of viscosity measurements (Figure 1C). These features of the TUBPV indicate great potential in point-of-care blood testing.

**FIGURE 2:**
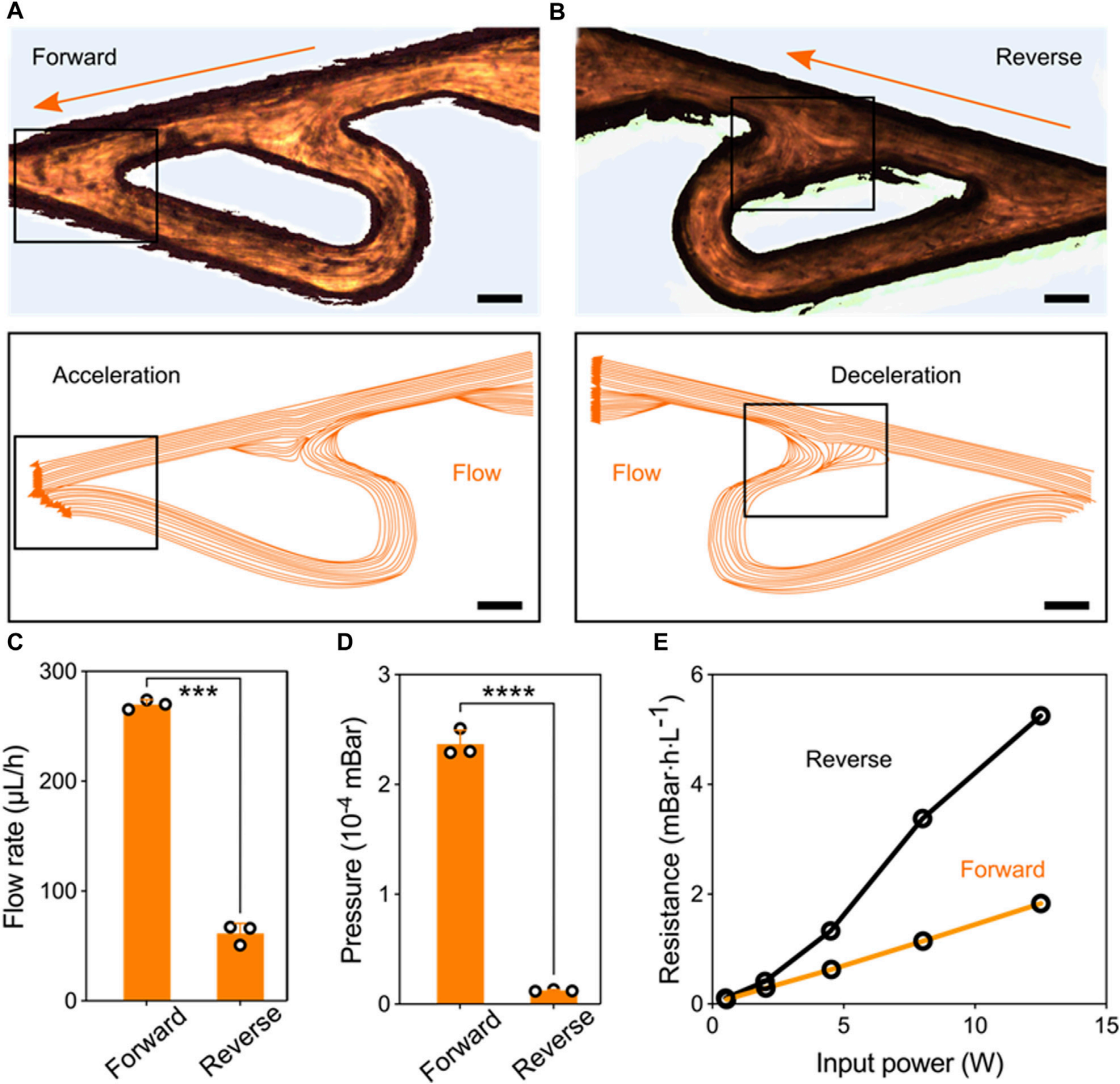
Performance of the Tesla valve’s conduit. **(A)** Streakline visualization of the forward flow accelerated by the Tesla valve’s structure (Scale bar: 150 μm). **(B)** Streakline visualization of the reverse flow decelerated by the Tesla valve’s structure (Scale bar: 150 μm). **(C)** Quantification of the forward and reverse flow rate pumped by the fixed ultrasonic pressure. **(D)** Quantification of the pressure variation at forward and reverse flowing directions. **(E)** Hydrodynamic resistance *versus* input signal power for the forward and reverse flowing directions.

**Performance of the Tesla valve’s conduit.** To access the regulation of the Tesla valve conduit on the liquid flows, we introduced liquids in the forward and reverse directions, respectively (Figures 2A,B). The relative streakline visualization reflected the effects of the conduit structures on flow acceleration and deceleration (Supplementary Movie S2 and Supplementary Movie S3). Using the same pressure as liquid propulsion, flow rates under two directions inside the conduit were significantly different (Figure 2C). The equivalent pressure values also indicated that the unique structures of Tesla valve have diodic behavior (Figure 2D). Besides, we quantified the hydrodynamic resistance R=∆P/Q *versus* input signal power for the forward and reverse flowing directions: ∆P is the pressure difference, Q is the volumetric flow rate. Figure 2E showed the larger resistance on the reverse flowing directions. These results indicate that the Tesla-like channel can convert high-frequency pulsatile flows into unidirectional flows that towards the forward direction.

**FIGURE 3:**
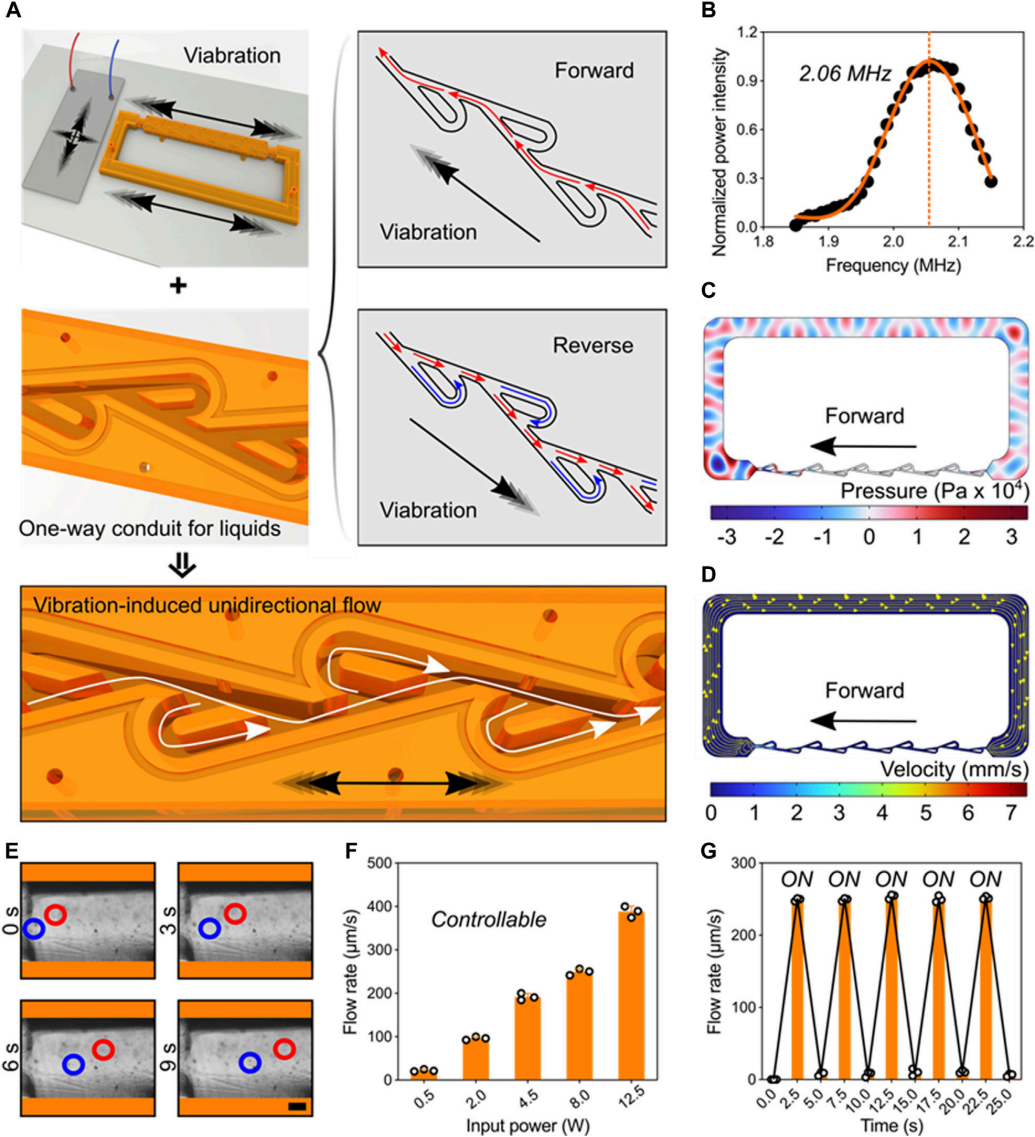
Pumping behavior powered by ultrasonic-structure interactions. **(A)** Schematics of one-way liquid conduit driven by ultrasonic vibration and regulated by Tesla valve’s diodicity. **(B)** The working frequency of the piezoelectric transducer. **(C)** Acoustic pressure field in the Tesla-like channel. **(D)** Numerical simulation showing the flow direction inside the channel. **(E)** Experimental images showing the unidirectional pumping behavior in the channel by indicating the motions of polystyrene beads at different time points. Red and blue circles represent two typical groups of microbeads (Scale bar: 250 μm). **(F)** The pumping flow rate can be precisely controlled by tuning the input signal power. **(G)** The programming pumping behavior.

**Pumping behavior powered by ultrasonic-structure interactions.** We next tested and validated the unidirectional pumping powered by the ultrasonic-structure interactions using theoretical and experimental approaches. Here, the piezoelectric transducer and Tesla valve conduit were deposited on the glass substrate. Applying electric signals on the transducer, high-frequency vibrations can be generated and induce the forward and reverse pulsatile flows inside the channel. Owing the fluidic diode property of Tesla valve, the liquid pressure in reverse direction is greatly larger than that in forward direction. Consequently, stable unidirectional flow can be generated in the ultrasonic-coupled structures (Figure 3A). Under the resonant frequency, we performed the numerical simulation that showed the piezoelectric transducer can drive one-way flow inside Tesla-like channel (Figures 3B–D). Flow details in basic unit of Tesla valve showed the fluidic diodic behavior (Supplementary Figure S2). To assess the unidirectional flow, we added polystyrene beads in the liquid and recorded their movements at different time points (Figure 3E). Further, the flow rate can be precisely controlled by the input signal power, ranging from tens to hundreds of micrometers per seconds (Figure 3F). In particular, the pumping performance was programmable and repeatable by using cyclic signal stimulus (Figure 3G and Supplementary Movie S4). Thus, the digital and controllable ultrasonic stimulus ensures the reliable and stable driving force to the one-way liquid movement.

**FIGURE 4:**
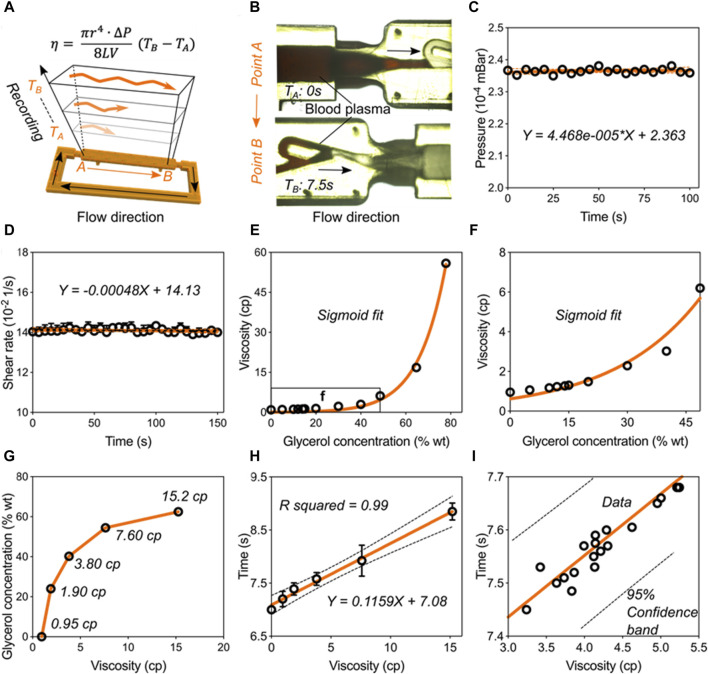
Validation of the blood viscometer capacity. **(A)** Schematics showing the working mechanism of blood viscometer. η-viscosity, which is proportional to the flowing time from position A to B in the channel. r -the channel radius, ∆P -the pressure inside the channel, L -the length from position A to B, V -the liquid volume. **(B)** Typical images showing the recording of flows from position A to **(B) (C)** Pressure variation over time. **(D)** Shear rate variation over time. **(E)** Fitting curve of glycerol’s viscosity at different concentrations. **(F)** The enlarged fitting curve of glycerol’s viscosity at low concentrations. **(G)** The relative concentrations of glycerol solution at different viscosities, which are used to calibrate the blood viscometer. **(H)** Calibration of the blood viscometer. A representative plot showing the mathematical relationship between viscosity and flowing time. **(I)** Verification of the prediction accuracy of the liquid viscometer.

**Validation of the blood viscometer capacity.** Having validated the pumping behavior in the Tesla-like channel under the steady ultrasonic driving force, we next calibrated and verified the capacity of this ultrasound waves-powered Tesla-like channel on liquid viscosity measurements. Based on the Hagen-Poiseuille Equation 1, liquid viscosity is proportional to the flowing time from position A to B, owing to other parameters can be regarded as the constant factor (Figures 4A,B). By applying an 8-w power of electric signal on the piezoelectric transducer, steady pressure can be obtained inside the Tesla-like channel (Figure 4C). We found that the temperature in the channel was independent from the ultrasonic radiation (Supplementary Figure S3). Besides, the shear rate remained constant due to the stable flow rate, as shown in Figure 4D, indicating the accuracy on measuring liquid viscosities. Benefitting from the reliable and stable pumping performance, the ultrasound waves-powered Tesla-like channel herein acted as a proof of concept for the liquid viscosity measurement.

The calibration and verification steps need to be carried out for transforming the ultrasound waves-powered Tesla-like channel into practical viscometer. We used glycerol solutions to calibrate the viscometer performance (Figures 4E,F). When viscosity values of glycerol solutions were plotted against their concentrations, we found that they followed a sigmoidal trend. Next, five standard glycerol solutions with viscosities at 0.95 cp, 1.90 cp, 3.80 cp, 7.60 cp, and 15.2 cp were prepared following the sigmoidal fit and added into the channel (Figure 4G). Based on the relative flowing time recording, we obtained the mathematical formulation of liquid viscosity and flowing time, as shown in Figure 4H. We then experimentally tested this model by investigating flowing times of twenty kinds of liquids (Figure 4I). All the liquids data were presented in Supplementary Table S1. We found that all the results were located in the 95% confidence band of the formulation. These results indicated that the ultrasound waves-powered Tesla-like channel can serve as practical viscometer with high stability and accuracy.

**FIGURE 5:**
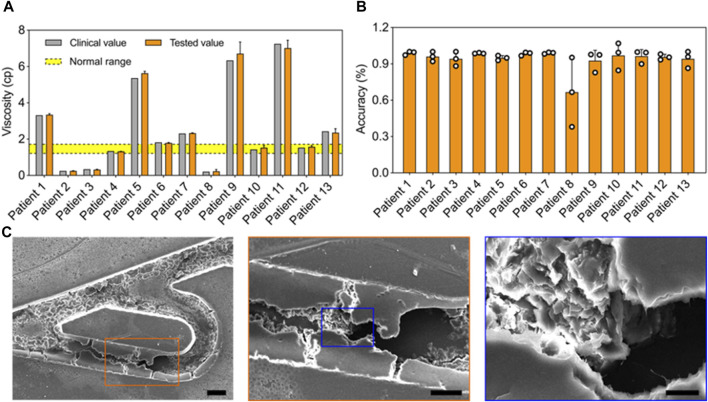
Rapid hemodynamic analysis *in vitro*. **(A)** Comparison of the viscosity values between clinical and this viscometer’s results. The normal viscosity value ranges from 1.26 to 1.7 cp. **(B)** The accuracy result of the blood viscometer referring to 13 patient groups. The average accuracy is 95.4%. **(C)** The typical scanning electron micrographs showing the coagulation result.

**Rapid hemodynamic analysis *in vitro*.** To test the practical performance of the Tesla valves and ultrasound waves-powered blood plasma viscometer (TUBPV), we analyzed the hemodynamics including viscosity measurements of blood plasma samples and the blood coagulation monitoring. First, we measured the blood plasma viscosities by assessing the flowing time from position A to B in the empty channel (Figure 5A). Our TUBPV achieved the rapid measurement of viscosity within 10 s for each sample, avoiding the platelet aggregation. In particular, our TUBPV can distinguish the blood states in a precise manner. Figure 5B showed the accuracy of our TUBPV at 95.4% on the viscosity measurement compared with the clinical measured values. Besides, our TUBPV can monitor the coagulation status of patients in real time. Owing to the microscale and vascular-like channel, coagulation can form after long-time closed-loop flowing, as shown in Figure 5C. Collectively, our TUBPV provides a new type of viscometer that enables the precise measurement of blood plasma viscosity and the real-time monitoring of blood coagulation status simultaneously.

## Discussion and conclusion

We have demonstrated a novel viscometer that offers the stable, fast, disposable, small-volume, and precise measurement of blood plasma’s viscosity, providing a point-of-care method for precisely monitoring blood hemodynamics. This viscometer exploits Tesla-like flow regulator, ultrasound-based liquid pumping, and on-chip miniaturization to achieve both real-time viscosity measurements and hemodynamic analysis *in vitro*. This viscometer can be adapted for viscosity analysis of a range of biological fluids with the rapid feature and clear clinical implications.

The performance and capability of this Tesla valves and ultrasound waves-powered blood plasma viscometer (TUBPV) can be further improved and extended. The dimension of the Tesla-like channel could be optimized for obtaining an optimal ability in flow regulation, resulting in a more stable flow and less energy consumption. Besides, the structure and unit number of the Tesla-like channel has a great influence on the interaction with ultrasound waves. The simulated and experimental strategies could be used to improve the efficiency of ultrasound energy converted to flow motions. Furthermore, integrating miniaturized power sources, signal generator, and imaging apparatus would significantly enhance viscometer portability and practicality, such as enabling the quantification of plasma coagulation under fluid flow. Leveraging the integrated viscometer system could promote the point-of-care applications in clinic.

## Methods

**Fabrication of Tesla-like channel**. This channel was fabricated by using maskless UV lithography (TuoTuo Technology, China) with 40 μm of layer resolution. The photocurable resin (HTL RESIN) was the functional acrylate, photo-initiator, and crosslinker, which was crosslinkable under the exposure of 405 nm light. The whole Tesla valve conduit was set as 3.5 cm × 1 cm × 0.2 cm. The length from position A to B in Tesla-like channel was 1.8 cm. There were one inlet and one outlet for introducing liquids. The channel was designed by Cinema 4D software and exported as stereolithography (STL) file. The channel file then was loaded into 3D printer (TTT-07-UV Litho-ACA) with 40 μm of layer resolution.

**Activation of the Tesla valves and ultrasound waves-powered blood plasma viscometer**. This viscometer was driven by sine radio frequency (RF) signals (working frequency, 2.06 MHz; power, 0–12.5 w) to achieve the stable flow pumping. The electric signals were controlled by a function generator (DG1022, RIGOL, China) and a power amplifier (LZY-22+, Mini-circuit, United States). By the electric stimulus, the piezoelectric transducer (PZT, Shenlei Ultrasonics, China) can generate ultrasound waves and propagated through the glass slice, and eventually interacted with the structures inside Tesla-like channel. Th high-frequency pulsatile flows can be generated in the conduit. With the regulation of the Tesla-like channel, pulsatile flows can be converted into unidirectional flows that towards the forward direction.

**Simulation of the ultrasonic-powered pumping behavior**. The pumping behavior was simulated via the finite element analysis (COMSOL 6.0, COMSOL Inc., Burlington, MA United States). The details can be found in the Supplementary Methods.

**Preparation of Glycerol solutions**. Five standard glycerol (G5516, Sigma Aldrich) solutions with viscosities at 0.95 cp, 1.90 cp, 3.80 cp, 7.60 cp, and 15.2 cp were prepared for calibrating the viscometer. Their concentrations were 0, 24, 40.2, 54.5, 62.5 % wt. All the solutions were prepared and used under the room temperature.

**Preparation of blood plasma samples for viscosity measurements**. Human blood plasma was obtained from Nanjing Drum Tower Hospital. All the experiments were conducted following the clinical guidelines of the Medical Clinical Research Ethics Review Form of Nanjing Drum Tower Hospital. This study was approved by the Institutional Review Board (IRB) of Nanjing Drum Tower Hospital (2022-481), Nanjing, China. Briefly, the plasma samples were used at the same time on repeated testing via commercial fully automatic hemorrheometer (Precil, ZL9 100C, China) and our viscometer. The volumes of plasma samples used in the commercial machine and our viscometer were 5 mL and 0.2 mL, respectively. Notably, Na salt at 4 mmol/L blood plasma was used as the anticoagulant, ensuring the minimal change in the plasma viscosity. All the samples were tested at the room temperature.

**Ethics approval and consent to participate**. This study was carried out using the blood samples and their information of 13 patients from our hospital. This study was approved by the Institutional Review Board (IRB) of Nanjing Drum Tower Hospital (approval no. 2022-481), Nanjing, China. Participants provided written informed consent prior to taking part in the study.

**Characterizations**. Movies were recorded by using an IX83 microscope (Olympus, Japan) with a CCD camera (DP72, Olympus, Japan). The imaging interval time of the CCD we used was 30 ms. Optical photos were obtained from a Leica stereomicroscope (M205FA, Germany). High-resolution images were acquired from a scanning electron microscope (JSM-IT200, Japan). The streakline visualization was performed in software ImageJ (Wayne Rasband, NIH). The viscosity of glycerol solutions was plotted and fitted using GraphPad Prism 10. We characterized the status of blood coagulation utilizing hydrodynamic principles, establishing a criterion whereby flow rates below 5 μL/h indicate a significant coagulation event. This decrease in flow rate is attributed to the obstruction of the microfluidic channel by coagulated blood samples. Moreover, the influence of acoustic waves on the process of blood coagulation was standardized across all experiments through the use of a consistent acoustic signal. Consequently, this standardization allows for the exclusion of ultrasound effects from the final comparative analysis of the coagulation outcomes.

**Statistical analysis**. The data of two comparing groups were analyzed by using the two-tailed *t*-test in GraphPad Prism 10. The statistical significance was denoted as: *p<0.05,**p<0.01,***p<0.005,****p<0.001 .

## Data Availability

The original contributions presented in the study are included in the article/Supplementary Material, further inquiries can be directed to the corresponding authors.
