# Supplementary material for: A bio-fabricated tesla valves and ultrasound waves-powered blood plasma viscometer
Source: Front Bioeng Biotechnol. 2024 Apr 24;12:1394373. doi: 10.3389/fbioe.2024.1394373 (PMC11076727; doi:10.3389/fbioe.2024.1394373)
Supplement: Supplementary file 5 [file DataSheet1.docx]

**Supplementary**

**A bio-fabricated tesla valves and ultrasound waves-powered blood plasma viscometer**

Wenqin Chen^1^, Mao Xia^1^, Wentao Zhu^2^, Zhiye Xu^1,^*, Bo Cai^2,^*, Han Shen^1,^*

*^1^ Department of Clinical Laboratory, Nanjing Drum Tower Hospital, Affiliated Hospital of Medical School, Nanjing University, Nanjing 210008, China*

*^2^ School of Environment and Health, Jianghan University, Wuhan 430056, China*

* Corresponding author.

E-mail addresses: xuzhiye@sina.com (Z. Xu), [bcai@jhun.edu.cn](mailto:bcai@jhun.edu.cn) (B. Cai), [shenhan@njglyy.com](mailto:shenhan@njglyy.com) (H. Shen).

**List of Contents**

**Supplementary Methods**

Simulations for pumping behavior.

**Supplementary Figures**

**Supplementary Fig. 1:** The Tesla valves and ultrasound waves-powered blood plasma viscometer (TUBPV).

**Supplementary Fig. 2:** Simulation modeling of the acoustic pumping behavior.

**Supplementary Fig. 3:** Temperature variation over time.

**Supplementary Fig. 4:** Blood plasma samples from patients.

**Supplementary Table**

Patient information.

**Supplementary Movies**

**Supplementary Movie 1:** The flow powered by ultrasound waves.

**Supplementary Movie 2:** The forward flow behavior in the Tesla-like channel.

**Supplementary Movie 3:** The reverse flow behavior in the Tesla-like channel.

**Supplementary Movie 4:** The programmable flow controlled by ultrasonic stimulus.

**Supplementary Methods**

The simulation is performed under the software COMSOL Multiphysics 6.1. In a mathematical sense, where the acoustic field is the linearized first-order field of the Navier–Stokes equations, the acoustic streaming field is the time-averaged second-order field in the perturbation scheme. Briefly, acoustic streaming is a steady fluid flow induced by the ultrasound waves-structure interaction because of nonlinear terms in the Navier-Stokes equations:

$$\begin{aligned} \rho\left( \frac{\partial\boldsymbol{u}}{\partial t}+\boldsymbol{u}\cdot\nabla\boldsymbol{u} \right)=-\nabla p+\rho\boldsymbol{g}+\mu\nabla^{2}\boldsymbol{u}\#\left( 2 \right) \end{aligned}$$

where relevant parameters are: $\rho$, the density; $\boldsymbol{u}$, the velocity; $p$, the pressure; $\boldsymbol{g}$, the gravitational constant; and μ, the dynamic viscosity.

Acoustic domain forces are simulated and applied to the 'Laminar Flow' interface through a multiphysics feature known as 'Acoustic Streaming Domain Coupling.' This feature couples the 'Thermoviscous Acoustics' and 'Frequency Domain' interfaces with the 'Laminar Flow' interface. Although there is a complementary 'Acoustic Streaming Boundary Coupling' available for both 'Pressure Acoustics' and 'Frequency Domain' in 'Thermoviscous Acoustics', it remains unused in this specific setup due to its negligible contributions. Including it would be overshadowed by the dominance of the primary feature. It's worth noting that the boundary coupling from thermoviscous acoustics becomes significant only when the boundary exhibits vibrations. Therefore, if the vibrations of the thin flaps were included in the model, the 'Acoustic Streaming Boundary Coupling' would be essential for accurately simulating the resulting steady fluid flow.

**Supplementary Figures**

**Supplementary Fig. 1.** **The Tesla valves and ultrasound waves-powered blood plasma viscometer (TUBPV).** This viscometer includes a piezoelectric transducer and a 3D-printed channel with Tesla valve structures.

**Supplementary Fig. 2. Simulation modeling of the acoustic pumping behavior.** (**a**) Geometry of the Tesla-like channel pumped by ultrasound waves. (**b**) Numerical simulation showing the enlarged flow behavior near the Tesla valve structure.

**Supplementary Fig. 3. Temperature variation over time.** The temperature in the channel was independent from the ultrasonic radiation.

**Supplementary Fig. 4. Blood plasma samples from patients.** All the blood sample were used at the same time point for precise viscosity measurements.

**Supplementary Table**

| Liquid-1  4.1345 cp  7.55 s | Liquid-2  5.2134 cp  7.68 s | Liquid-3  4.9563 cp  7.65 s | Liquid-4  4.2353 cp  7.57 s | Liquid-5  5.0045 cp  7.66 s | Liquid-6  3.7342 cp  7.51 s | Liquid-7  4.1435 cp  7.56 s | Liquid-8  4.1435 cp  7.56 s | Liquid-9  4.1345 cp  7.55 s | Liquid-10  4.2543 cp  7.57 s |
| --- | --- | --- | --- | --- | --- | --- | --- | --- | --- |
| Liquid-11  5.2345 cp  7.68 s | Liquid-12  4.6234 cp  7.61 s | Liquid-13  3.8654 cp  7.52 s | Liquid-14  3.8345 cp  7.51 s | Liquid-15  4.1345 cp  7.55 s | Liquid-16  3.4210 cp  7.47 s | Liquid-17  4.2130 cp  7.56 s | Liquid-18  3.6343 cp  7.50 s | Liquid-19  5.2436 cp  7.68 s | Liquid-20  3.2412 cp  7.45 s |

**Supplementary Table 1. Viscosity detail of validation liquids.**

All the liquid samples tested in the viscometer were glycerol solution.

| Blood donor | Gender | Age | Disease condition | Clinical viscosity value |
| --- | --- | --- | --- | --- |
| Sample 1 | Female | 56 | Vaginitis | 3.32 |
| Sample 2 | Female | 39 | Irregular menstruation | 0.25 |
| Sample 3 | Female | 31 | Abnormal coagulation function | 0.34 |
| Sample 4 | Female | 33 | Thrombocytopenia | 1.34 |
| Sample 5 | Male | 73 | Chronic kidney disease stage 5 | 5.36 |
| Sample 6 | Female | 87 | Peritoneal dialysis | 1.82 |
| Sample 7 | Female | 30 | Severe preeclampsia | 2.31 |
| Sample 8 | Female | 30 | Abnormal coagulation function | 0.21 |
| Sample 9 | Female | 38 | Chronic kidney disease stage 5 | 6.34 |
| Sample 10 | Female | 30 | Normal | 1.43 |
| Sample 11 | Female | 35 | Chronic kidney disease stage 5 | 7.25 |
| Sample 12 | Female | 34 | Normal | 1.53 |
| Sample 13 | Female | 36 | Sigmoid colon perforation | 2.43 |

**Supplementary Table 2.** **Patient information.**
